# BAFF/APRIL expression-guided telitacicept therapy demonstrates superior efficacy in systemic lupus erythematosus patients: a real-world comparative study

**DOI:** 10.3389/fmed.2025.1608085

**Published:** 2025-08-22

**Authors:** Li Huang, Yingzi Huang, Jiamin Zeng, Huhai Zhang, Liping Chen, Yi Li, Hongwen Zhao, Xiaopeng Tang

**Affiliations:** Nephrology Department, The First Affiliated Hospital of Army Medical University, Chongqing, China

**Keywords:** systemic lupus erythematosus, BAFF, APRIL, telitacicept, belimumab, personalized medicine, biomarker-guided therapy

## Abstract

**Background:**

Biological agents targeting B-cell pathways represent significant advances in systemic lupus erythematosus (SLE) management, yet optimal patient selection remains challenging. This study evaluated whether BAFF/APRIL expression testing could guide personalized treatment decisions in SLE patients.

**Methods:**

In this real-world observational study, we compared two treatment strategies in 86 SLE patients: personalized therapy with telitacicept in BAFF/APRIL double-positive patients (*n* = 14) versus conventional belimumab therapy without expression testing (*n* = 72). Clinical responses, laboratory parameters, and steroid-sparing effects were assessed at 3 and 6 months.

**Results:**

Despite having significantly higher baseline disease activity (SLEDAI 18.79 ± 9.34 vs. 8.86 ± 4.3) and more severe proliferative lupus nephritis (Class IV/IV + V in 78.6% vs. 52.8%), the BAFF/APRIL-guided telitacicept group achieved higher complete response rates (57.1% vs. 48.6%) and lower non-response rates (7.1% vs. 23.6%) compared to the conventional belimumab group. The BAFF/APRIL-guided group showed more substantial improvements in complement C3 levels (Δ = 0.49 vs. 0.24, *p* < 0.001) and anti-dsDNA antibody reduction (Δ = −177.07 vs −117.00, *p* = 0.028) at 6 months. Notably, normalization of immunological parameters was significantly better in the personalized therapy group, with dsDNA abnormalities decreasing from 92.9 to 7.1% (vs 91.7–32.5%) and C3 abnormalities from 100 to 28.6% (vs 91.7–61.1%). The BAFF/APRIL-guided group also achieved greater steroid dose reduction at 6 months (Δ = −35.00 vs −25.00 mg, *p* = 0.014).

**Conclusion:**

BAFF/APRIL expression-guided telitacicept therapy demonstrated superior efficacy in improving clinical responses and laboratory parameters in SLE patients compared to conventional belimumab therapy, despite treating patients with more severe baseline disease. This real-world study provides preliminary evidence supporting BAFF/APRIL testing as a potential biomarker-driven approach for personalized SLE management, warranting further prospective validation.

## Introduction

Systemic lupus erythematosus (SLE) is a heterogeneous and complex autoimmune disease characterized by dysregulated immune responses leading to multiorgan involvement and significant morbidity. Despite advances in therapeutic approaches, many patients continue to experience persistent disease activity, organ damage, and impaired quality of life, highlighting the need for more effective and personalized treatment strategies (1).

B cells play a central role in SLE pathogenesis through autoantibody production, cytokine secretion, antigen presentation, and immune dysregulation (2). Among the key regulators of B cell biology, two members of the tumor necrosis factor (TNF) superfamily—B cell activating factor (BAFF, also known as BLyS) and a proliferation-inducing ligand (APRIL)—have emerged as significant therapeutic targets (3, 4). BAFF binds to three receptors: BAFF receptor (BAFF-R), transmembrane activator and CAML interactor (TACI), and B cell maturation antigen (BCMA), while APRIL binds to TACI and BCMA (5). Both cytokines promote B cell survival, differentiation, and antibody production, with elevated serum levels commonly observed in SLE patients (6, 7).

The clinical relevance of the BAFF pathway in SLE has been established through the successful development of belimumab, a monoclonal antibody targeting soluble BAFF, which became the first approved biologic for SLE treatment (8). However, response rates to belimumab remain suboptimal, with approximately 40–60% of patients achieving clinically meaningful improvement (9, 10). This limited efficacy may be partly attributed to the continued activity of APRIL, which can sustain autoimmune B cell survival through shared receptor pathways even when BAFF is neutralized (11).

Telitacicept (RC18, trade name Tai’ai®) represents a novel therapeutic approach that simultaneously targets both BAFF and APRIL pathways. Telitacicept received conditional marketing approval from China’s National Medical Products Administration (NMPA) in March 2021 for the treatment of active SLE, followed by full marketing approval in November 2023. The drug was subsequently included in China’s National Reimbursement Drug List (NRDL) in December 2021, ensuring broader patient accessibility. Since its initial SLE approval, telitacicept has also received marketing approval in China for rheumatoid arthritis (July 2024) and generalized myasthenia gravis (May 2025). This recombinant fusion protein consists of the extracellular domain of TACI receptor linked to the Fc portion of human IgG1, enabling it to bind and neutralize both BAFF and APRIL (12). Recent clinical trials have demonstrated the efficacy of telitacicept in reducing SLE disease activity with an acceptable safety profile (13, 14). The dual inhibition mechanism potentially offers advantages over single-cytokine targeting strategies by providing more comprehensive suppression of pathogenic B cell responses (15).

Despite these advances, biomarker-guided therapeutic approaches remain underdeveloped in SLE management. Unlike oncology, where molecular profiling routinely informs treatment decisions, SLE therapy selection typically relies on clinical phenotypes rather than underlying biological mechanisms (16). This approach fails to address the heterogeneity of SLE pathogenesis and may partly explain the modest efficacy of current treatments.

The expression levels of BAFF and APRIL may hold particular promise as predictive biomarkers for response to targeted therapies. Previous studies have shown that elevated serum BAFF levels correlate with disease activity in SLE (17), while tissue expression of BAFF/APRIL in lupus nephritis biopsies may reflect local B cell activation and inflammation (18). However, the clinical utility of BAFF/APRIL expression testing in guiding therapeutic decisions remains largely unexplored.

In this real-world observational study, we investigated whether a personalized treatment approach based on BAFF/APRIL expression testing could enhance therapeutic outcomes in SLE patients. Specifically, we compared the clinical response, laboratory parameter improvement, and steroid-sparing effects between two treatment strategies: personalized therapy with telitacicept in patients with confirmed BAFF/APRIL double-positive expression versus conventional therapy with belimumab without expression testing. This study represents one of the first attempts to implement a biomarker-driven approach to SLE treatment selection in clinical practice and may provide preliminary evidence for a more individualized treatment paradigm in managing this challenging disease.

## Methods

### Study design

This was a real-world observational study conducted at the First Affiliated Hospital of Army Medical University, Chongqing, China between June 2023 and June 2024. The study compared two treatment strategies in patients with SLE: a personalized therapy approach using telitacicept for patients with confirmed BAFF/APRIL double-positive expression versus conventional therapy with belimumab without prior expression testing. The study protocol was approved by the Institutional Ethics Committee of the First Affiliated Hospital of Army Medical University (approval number: (B)KY2025111), and all patients provided written informed consent before participation. This study was conducted in accordance with the Declaration of Helsinki and Good Clinical Practice guidelines.

### Patients

A total of 86 patients with SLE were included in this study. All patients fulfilled the 2019 European League Against Rheumatism/American College of Rheumatology (EULAR/ACR) classification criteria for SLE. Inclusion criteria included: (1) Age ≥18 years; (2) Biopsy-proven lupus nephritis with renal biopsy performed within 6 months prior to enrollment; (3) Active disease defined as SLEDAI-2K score ≥4; (4) Inadequate response to standard therapy including corticosteroids and/or immunosuppressive agents. All patients in both treatment groups had lupus nephritis and available renal biopsy specimens for histopathological evaluation. For patients in the BAFF/APRIL-guided telitacicept group, renal biopsy specimens were additionally assessed for BAFF and APRIL expression, and only patients with double-positive expression for both markers were included in this treatment arm.

Patients were allocated to one of two treatment groups. The BAFF/APRIL-guided telitacicept group (*n* = 14) comprised patients who tested positive for both BAFF and APRIL expression in renal biopsy specimens and subsequently received telitacicept treatment. The conventional belimumab group (*n* = 72) included patients who received belimumab without prior BAFF/APRIL expression testing.

Key exclusion criteria included: severe active central nervous system lupus, severe active lupus nephritis requiring cyclophosphamide therapy, pregnancy or breastfeeding, active severe infection, history of malignancy within 5 years, and previous treatment with B cell-depleting therapies within 6 months prior to enrollment.

All patients continued to receive standard therapy, including corticosteroids, antimalarials, and immunosuppressive agents as clinically indicated. For the telitacicept group, patients received 160 mg administered subcutaneously once weekly. For the belimumab group, patients received 10 mg/kg administered intravenously every 2 weeks for the first 3 infusions and every 4 weeks thereafter.

### BAFF/APRIL expression testing

For patients in the telitacicept group, BAFF and APRIL expression was assessed in renal biopsy specimens using immunohistochemistry. Tissue sections were stained with anti-BAFF and anti-APRIL antibodies according to the manufacturer’s protocol. Expression was evaluated by two independent pathologists blinded to clinical data. Positive expression was defined as moderate to strong staining intensity in >10% of glomerular and/or tubulointerstitial cells. All patients in the telitacicept group demonstrated double-positive expression for both BAFF and APRIL. mTOR expression was also assessed using similar methodology.

### Histopathological assessment

Renal biopsy specimens were evaluated according to the International Society of Nephrology/Renal Pathology Society (ISN/RPS) classification for lupus nephritis. The activity index (AI) and chronicity index (CI) were calculated according to the National Institutes of Health (NIH) scoring system. Additional histopathological features assessed included mesangial proliferation, endothelial cell proliferation, crescent formation, inflammatory cell infiltration, wire loops, fibrinoid necrosis, and thrombotic microangiopathy (TMA).

### Outcomes and assessments

Patients were followed for 6 months after initiation of therapy. The primary outcome was clinical response at 6 months, categorized as complete response (CR), partial response (PR), or no response (NR). Complete response was defined as ≥50% reduction in SLEDAI-2K score, normalization of complement and anti-dsDNA antibody levels, urine protein <0.5 g/24 h, and prednisone dose ≤7.5 mg/day. Partial response was defined as ≥50% reduction in SLEDAI-2K score without meeting all criteria for CR. No response was defined as <50% reduction in SLEDAI-2K score or worsening of disease. Secondary outcomes included changes from baseline in laboratory parameters at 3 and 6 months (complement C3, anti-dsDNA antibodies, serum creatinine, serum albumin, 24-h urine protein, hemoglobin, and platelet count), proportion of patients with normalization of abnormal laboratory values at 3 and 6 months, and reduction in corticosteroid dose at 3 and 6 months. Safety assessments included monitoring for adverse events, serious adverse events, and infections, which were recorded throughout the study period.

### Statistical analysis

No formal sample size calculation was performed for this real-world observational study, which represents a limitation of the current research. The sample sizes were determined by the availability of patients meeting inclusion criteria and the feasibility of conducting BAFF/APRIL expression testing during the study period. Continuous variables are presented as mean ± standard deviation or median (interquartile range) as appropriate. Categorical variables are presented as numbers and percentages.

For baseline characteristics, comparisons between groups were performed using Student’s t-test or Mann–Whitney U test for continuous variables and Chi-square test or Fisher’s exact test for categorical variables, as appropriate.

For efficacy analyses, changes from baseline in continuous variables were compared between groups using Mann–Whitney U test due to the non-normal distribution of the data. The proportions of patients achieving clinical responses (CR, PR, NR) and normalization of laboratory parameters were compared using Chi-square test or Fisher’s exact test.

A *p*-value <0.05 was considered statistically significant. All statistical analyses were performed using SPSS version 25.0 (IBM Corp., Armonk, NY, USA) and R version 4.0.3 (R Foundation for Statistical Computing, Vienna, Austria).

## Results

### Patient characteristics

A total of 86 SLE patients were included in this study, with 14 patients in the BAFF/APRIL-guided telitacicept group and 72 patients in the conventional belimumab group. Demographic, clinical, and histopathological characteristics at baseline are presented in Table 1.

**Table 1:** Baseline demographic, clinical, and histopathological characteristics of patients with SLE.

Characteristics	Telitacicept group (*n* = 14)	Belimumab group (*n* = 72)
Age, years (Mean ± SD)	24.71 ± 9.43	32.75 ± 11.66
SLEDAI (Mean ± SD)	18.79 ± 9.34	8.86 ± 4.30
AI score (Mean ± SD)	8.21 ± 3.09	6.07 ± 3.83
CI score (Mean ± SD)	1.64 ± 1.86	2.22 ± 1.73
Gender, *n* (%)		
Female	10 (71.4%)	66 (91.7%)
Male	4 (28.6%)	6 (8.3%)
Pathological type, *n* (%)		
II	0 (0%)	4 (5.6%)
III	1 (7.1%)	16 (22.2%)
III + V	2 (14.3%)	3 (4.2%)
IV	7 (50.0%)	22 (30.6%)
IV + V	4 (28.6%)	16 (22.2%)
V	0 (0%)	5 (6.9%)
NA	0 (0%)	6 (8.3%)
Mesangial hyperplasia, *n* (%)		
Mild	3 (21.4%)	16 (22.2%)
Mild to moderate	6 (42.8%)	19 (26.4%)
Moderate	1 (7.1%)	8 (11.1%)
Moderate to severe	4 (28.6%)	11 (15.3%)
NA	0 (0%)	18 (25.0%)
Endothelial hyperplasia, *n* (%)		
Positive	14 (100%)	46 (63.9%)
Negative	0 (0%)	8 (11.1%)
NA	0 (0%)	18 (25.0%)
Crescents, *n* (%)		
Positive	11 (78.6%)	27 (37.5%)
Negative	3 (21.4%)	27 (37.5%)
NA	0 (0%)	18 (25.0%)
Inflammatory cell infiltration, *n* (%)		
Focal	8 (57.1%)	35 (48.6%)
Multifocal	6 (42.9%)	19 (26.4%)
NA	0 (0%)	18 (25.0%)
Wire loops, *n* (%)		
Positive	7 (50%)	11 (15.3%)
Negative	7 (50%)	43 (59.7%)
NA	0 (0%)	18 (25.0%)
Fibrinoid necrosis, *n* (%)		
Positive	3 (21.4%)	4 (5.6%)
Negative	11 (78.6%)	50 (69.4%)
NA	0 (0%)	18 (25.0%)
TMA, *n* (%)		
Positive	1 (7.1%)	1 (1.4%)
Negative	13 (92.9%)	53 (73.6%)
NA	0 (0%)	18 (25.0%)
BAFF, *n* (%)		
Positive	14 (100%)	–
APRIL, *n* (%)		
Positive	14 (100%)	–
mTOR, *n* (%)		
Positive	14 (100%)	–
Reason for treatment, *n* (%)		
Initial treatment	11 (78.6%)	27 (37.5%)
Relapse	3 (21.4%)	45 (62.5%)
Pulse steroid therapy, *n* (%)		
Yes	1 (7.1%)	35 (48.6%)
No	13 (92.9%)	37 (51.4%)

Demographic, clinical, and histopathological characteristics of patients in the BAFF/APRIL-guided telitacicept group (*n* = 14) and conventional belimumab group (*n* = 72). Data are presented as mean ± standard deviation or number (percentage). SLEDAI = Systemic Lupus Erythematosus Disease Activity Index; AI, Activity Index; CI, Chronicity Index; NA, Not Available; TMA, Thrombotic Microangiopathy; BAFF, B cell Activating Factor; APRIL, A Proliferation-Inducing Ligand; mTOR, mammalian Target Of Rapamycin.

Patients in the BAFF/APRIL-guided telitacicept group were significantly younger than those in the conventional belimumab group (24.71 ± 9.43 vs. 32.75 ± 11.66 years). The telitacicept group had a higher proportion of male patients (28.6% vs. 8.3%). Notably, the telitacicept group demonstrated substantially higher disease activity at baseline, with mean SLEDAI scores of 18.79 ± 9.34 compared to 8.86 ± 4.3 in the belimumab group. The activity index (AI) was also higher in the telitacicept group (8.21 ± 3.09 vs. 6.07 ± 3.83), while the chronicity index (CI) was lower (1.64 ± 1.86 vs. 2.22 ± 1.73).

Regarding histopathological features, the telitacicept group exhibited more severe proliferative lupus nephritis, with class IV and IV + V nephritis accounting for 78.6% of cases compared to 52.8% in the belimumab group. The telitacicept group showed higher rates of pathological activity markers, including endothelial cell proliferation (100% vs. 63.9%), crescent formation (78.6% vs. 37.5%), multifocal inflammatory cell infiltration (42.9% vs. 26.4%), and wire loop lesions (50% vs. 15.3%). Fibrinoid necrosis was also more common in the telitacicept group (21.4% vs. 5.6%).

The majority of patients in the telitacicept group were newly diagnosed and receiving first-line treatment (78.6%), whereas the belimumab group had a higher proportion of patients with relapsing disease (62.5%). Only 7.1% of patients in the telitacicept group had received prior steroid pulse therapy, compared to 48.6% in the belimumab group.

All patients in the BAFF/APRIL-guided telitacicept group tested positive for both BAFF and APRIL expression, as well as mTOR expression, in renal biopsy specimens, which was the basis for their treatment allocation.

### Clinical response

**Figure 1:**
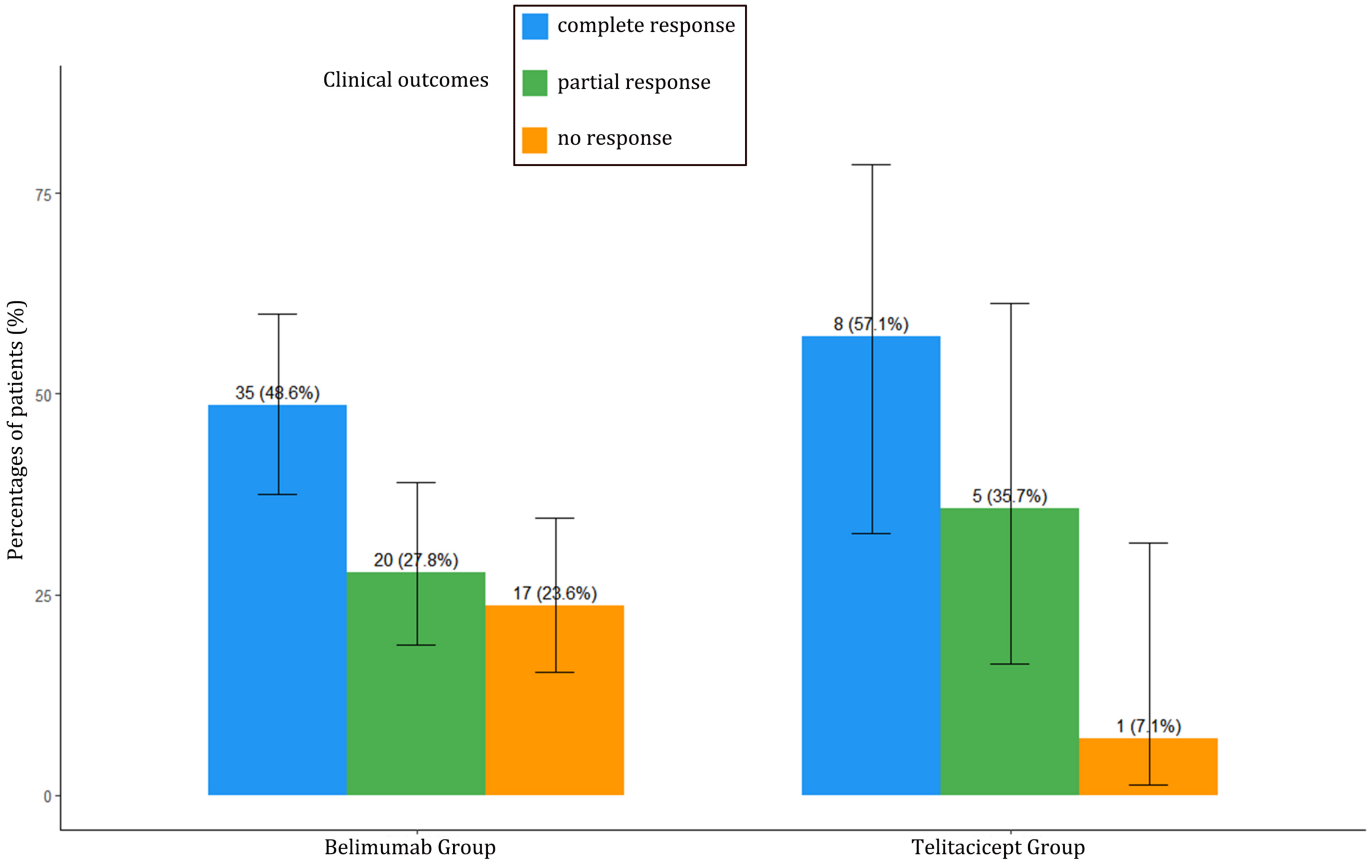
Clinical outcomes comparison between BAFF/APRIL-guided telitacicept group and conventional belimumab group at 6 months. Percentages of patients achieving complete response (CR), partial response (PR), or no response (NR) at 6 months in both treatment groups.

Despite having more severe baseline disease, the BAFF/APRIL-guided telitacicept group achieved better clinical outcomes at 6 months compared to the conventional belimumab group (Table 2). The telitacicept group demonstrated a higher complete response rate (57.1% vs. 48.6%) and partial response rate (35.7% vs. 27.8%), with a substantially lower non-response rate (7.1% vs. 23.6%) (Figure 1).

**Table 2:** Clinical outcome comparison between BAFF/APRIL-guided telitacicept group and conventional belimumab group.

Group	*N*	CR	PR	NR
		*n* %	*n* %	*n* %
Telitacicept group	14	8 (57.1)	5 (35.7)	1 (7.1)
Belimumab group	72	35 (48.6)	20 (27.8)	17 (23.6)

Clinical responses are categorized as Complete Response (CR), Partial Response (PR), or No Response (NR). CR was defined as ≥50% reduction in SLEDAI-2K score, normalization of complement and anti-dsDNA antibody levels, urine protein <0.5 g/24 h, and prednisone dose ≤7.5 mg/day. PR was defined as ≥50% reduction in SLEDAI-2K score without meeting all criteria for CR. NR was defined as <50% reduction in SLEDAI-2K score or worsening of disease.

### Laboratory parameters

**Figure 2:**
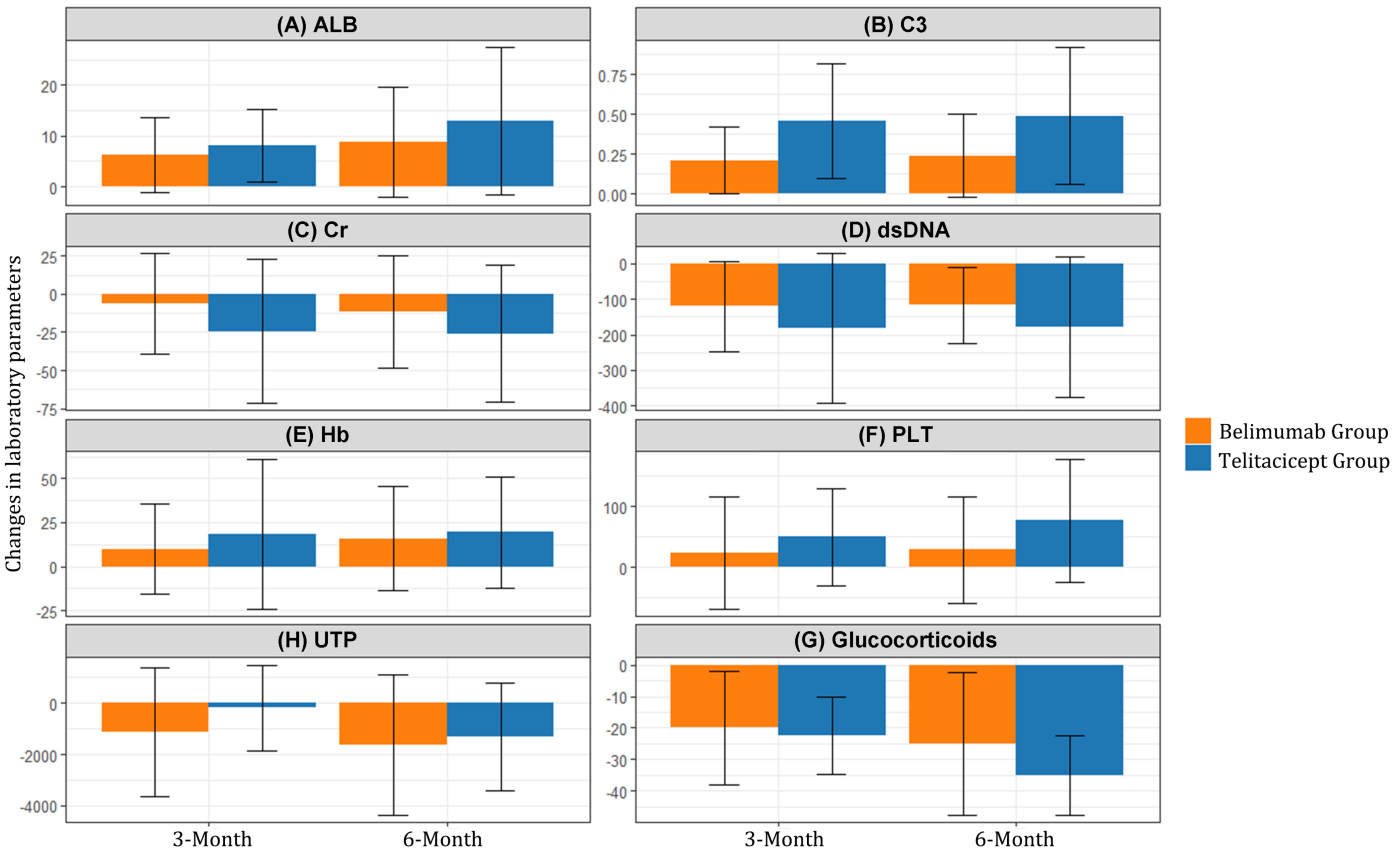
Changes in laboratory parameters from baseline to 3 and 6 months. Mean changes in **(A)** Serum albumin (ALB), **(B)** Complement C3, **(C)** Serum creatinine (Cr), **(D)** Anti-dsDNA antibodies, **(E)** Hemoglobin (Hb), **(F)** Platelet count (PLT), **(G)** 24-h urine protein (UTP), and **(H)** Glucocorticoids from baseline to 3 and 6 months in the BAFF/APRIL-guided telitacicept group (blue line) and conventional belimumab group (red line).

Changes from baseline in laboratory parameters at 3 and 6 months are summarized in Table 3 and illustrated in Figure 2. The telitacicept group showed significantly greater improvement in complement C3 levels at both 3 months (Δ = 0.46 vs. 0.21, *p* < 0.001) and 6 months (Δ = 0.49 vs. 0.24, *p* < 0.001) compared to the belimumab group. Similarly, the reduction in anti-dsDNA antibodies was more pronounced in the telitacicept group at 3 months (Δ = −181.66 vs. − 120.50, *p* = 0.015) and 6 months (Δ = −177.07 vs. − 117.00, *p* = 0.028).

**Table 3:** Changes from baseline in clinical indicators at 3 and 6 months.

Indicator	Time point	Telitacicept group	Belimumab group	*p*-value
C3	Δ 3 months	0.46 (0.36)	0.21 (0.21)	<0.001
	Δ 6 months	0.49 (0.43)	0.24 (0.26)	<0.001
dsDNA	Δ 3 months	−181.66 (211.38)	−120.50 (125.75)	0.015
	Δ 6 months	−177.07 (198.20)	−117.00 (106.75)	0.028
Cr	Δ 3 months	−24.25 (47.22)	−6.15 (32.99)	0.272
	Δ 6 months	−25.85 (44.75)	−11.62 (36.87)	0.054
ALB	Δ 3 months	8.10 (7.05)	6.35 (7.37)	0.416
	Δ 6 months	12.95 (14.50)	8.75 (10.85)	0.259
UTP	Δ 3 months	−172.50 (1,665.00)	−1,131.50 (2,520.75)	0.035
	Δ 6 months	−1,315.50 (2,117.00)	−1,632.00 (2,718.50)	0.854
Hb	Δ 3 months	18.50 (42.50)	10.00 (25.50)	0.436
	Δ 6 months	19.50 (31.50)	16.00 (29.50)	0.095
PLT	Δ 3 months	49.50 (80.00)	23.50 (92.00)	0.061
	Δ 6 months	77.00 (100.75)	28.50 (88.00)	0.044
Steroid dose	Δ 3 months	−22.50 (12.25)	−20.00 (18.12)	0.524
	Δ 6 months	−35.00 (12.50)	−25.00 (22.50)	0.014

Mean changes (standard deviation) from baseline in laboratory parameters and corticosteroid dose at 3 and 6 months in the BAFF/APRIL-guided telitacicept group and conventional belimumab group. Δ indicates change from baseline. *p*-values <0.05 are considered statistically significant. C3, Complement 3; dsDNA, double-stranded DNA antibody; Cr, Creatinine; ALB, Albumin; UTP, Urine Total Protein; Hb, Hemoglobin; PLT, Platelet count.

Serum albumin levels showed steady improvement in both groups, with a trend toward greater improvement in the telitacicept group at 6 months (Δ = 12.95 vs. 8.75, *p* = 0.259), although this difference did not reach statistical significance.

At 3 months, the telitacicept group showed a smaller reduction in 24-h urine protein compared to the belimumab group (Δ = −172.50 vs −1,131.50, *p* = 0.035). However, by 6 months, the urine protein reduction in the telitacicept group had improved substantially (Δ = −1,315.50), with no significant difference between groups (*p* = 0.854).

The telitacicept group demonstrated greater improvement in platelet count at 6 months (Δ = 77.00 vs. 28.50, *p* = 0.044) and hemoglobin levels showed a trend toward better improvement in the telitacicept group, though this did not reach statistical significance (Δ = 19.50 vs. 16.00, *p* = 0.095).

Notably, the telitacicept group achieved more substantial corticosteroid dose reduction at 6 months (Δ = −35.00 vs −25.00, *p* = 0.014) compared to the belimumab group, highlighting the enhanced steroid-sparing effect of this treatment approach (Figure 2).

### Normalization of laboratory parameters

**Figure 3:**
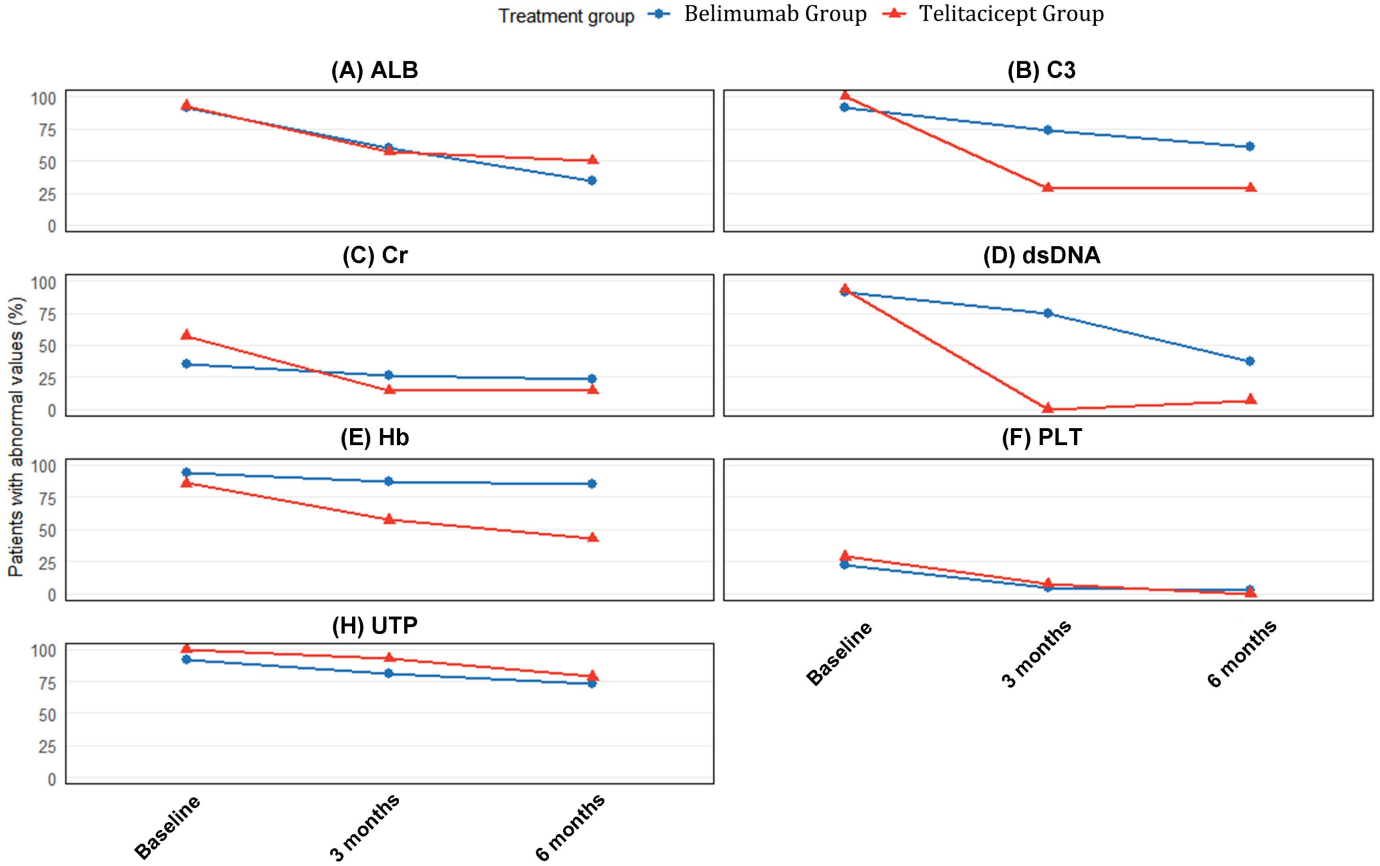
Proportion of patients with abnormal laboratory values at baseline, 3 months, and 6 months. Percentages of patients with abnormal values for **(A)** Serum albumin (ALB), **(B)** Complement C3, **(C)** Serum creatinine (Cr), **(D)** Anti-dsDNA antibodies, **(E)** Hemoglobin (Hb), **(F)** Platelet count (PLT), and **(G)** 24-h urine protein (UTP) at baseline (blue bars), 3 months (orange bars), and 6 months (gray bars) in the BAFF/APRIL-guided telitacicept group (Red panel) and conventional belimumab group (Blue panel). The values inside each bar represent the percentage of patients with abnormal values for each parameter.

The proportion of patients with abnormal laboratory values at baseline, 3 months, and 6 months is presented in Table 4 and visualized in Figure 3. The telitacicept group showed more rapid and complete normalization of immunological parameters compared to the belimumab group.

**Table 4:** Number of patients with abnormal laboratory values at baseline, 3 months, and 6 months.

Parameter	Time point	Telitacicept group (*N* = 14)	Belimumab group (*N* = 72)
C3 abnormal	Baseline	14 (100%)	66 (91.7%)
	3 months	4 (28.6%)	53 (73.6%)
	6 months	4 (28.6%)	44 (61.1%)
dsDNA abnormal	Baseline	13 (92.9%)	66 (91.7%)
	3 months	0 (0%)	54 (75.0%)
	6 months	1 (7.1%)	27 (32.5%)
Cr abnormal	Baseline	8 (57.1%)	25 (34.7%)
	3 months	2 (14.3%)	19 (26.4%)
	6 months	2 (14.3%)	17 (23.6%)
ALB abnormal	Baseline	13 (92.9%)	66 (91.7%)
	3 months	8 (57.1%)	43 (59.7%)
	6 months	7 (50.0%)	25 (34.7%)
UTP abnormal	Baseline	14 (100%)	66 (91.7%)
	3 months	13 (92.9%)	58 (80.6%)
	6 months	11 (78.6%)	53 (73.6%)
Hb abnormal	Baseline	12 (85.7%)	68 (94.4%)
	3 months	8 (57.1%)	63 (87.5%)
	6 months	6 (42.9%)	61 (84.7%)
PLT abnormal	Baseline	4 (28.6%)	16 (22.2%)
	3 months	1 (7.1%)	3 (4.2%)
	6 months	0 (0%)	2 (2.8%)

Number and percentage of patients with abnormal laboratory values at baseline, 3 months, and 6 months in the BAFF/APRIL-guided telitacicept group (*N* = 14) and conventional belimumab group (*N* = 72). C3, Complement 3; dsDNA, double-stranded DNA antibody; Cr, Creatinine; ALB, Albumin; UTP, Urine Total Protein; Hb, Hemoglobin; PLT, Platelet count.

Regarding immunological parameters, the proportion of patients with abnormal C3 decreased from 100% at baseline to 28.6% at both 3 and 6 months in the telitacicept group, compared to a reduction from 91.7% at baseline to 66.7% at 3 months and 61.1% at 6 months in the belimumab group. This superior normalization pattern is consistent with the significantly greater quantitative improvement in C3 levels observed at both timepoints (3 months: Δ = 0.46 vs. 0.21, *p* < 0.001; 6 months: Δ = 0.49 vs. 0.24, *p* < 0.001).

The improvement in anti-dsDNA antibodies was particularly striking in the telitacicept group, with the proportion of patients with abnormal values decreasing from 92.9% at baseline to 0% at 3 months and 7.1% at 6 months. In contrast, the belimumab group showed more modest improvement, with abnormal anti-dsDNA proportions decreasing from 91.7% at baseline to 58.3% at 3 months and 32.5% at 6 months.

For renal function parameters, differential improvement patterns were observed between groups. Serum creatinine showed more substantial quantitative improvements in the telitacicept group at both 3 months (Δ = −24.25 vs. −6.15, *p* = 0.272) and 6 months (Δ = −25.85 vs. −11.62, *p* = 0.054), with the 6-month difference approaching statistical significance. Correspondingly, the proportion of patients with abnormal serum creatinine in the telitacicept group decreased from 57.1% at baseline to 14.3% at both 3 and 6 months, compared to a reduction from 34.7% at baseline to 29.2% at 3 months and 23.6% at 6 months in the belimumab group.

The normalization of serum albumin levels showed comparable improvement patterns between groups. At 3 months, 64.3% of telitacicept patients and 59.7% of belimumab patients still had abnormal albumin levels, improving to 50.0 and 34.7%, respectively, at 6 months. This gradual normalization pattern aligns with the consistent but non-significant quantitative improvements observed in both groups (3 months: Δ = 8.10 vs. 6.35, *p* = 0.416; 6 months: Δ = 12.95 vs. 8.75, *p* = 0.259).

Regarding hematological parameters, the normalization of anemia showed progressive improvement in the telitacicept group, with the proportion of patients with abnormal hemoglobin decreasing from 85.7% at baseline to 57.1% at 3 months and 42.9% at 6 months. In contrast, the belimumab group showed more modest improvement from 94.4% at baseline to 88.9% at 3 months and 84.7% at 6 months. This differential improvement is supported by the quantitative hemoglobin changes (telitacicept vs. belimumab: 3 months Δ = 18.50 vs. 10.00, *p* = 0.436; 6 months Δ = 19.50 vs. 16.00, *p* = 0.095).

Platelet normalization was more effective in the telitacicept group, with complete normalization achieved by 6 months compared to 83.3% normalization in the belimumab group. This superior outcome is consistent with the significantly greater quantitative improvement at 6 months (Δ = 77.00 vs. 28.50, *p* = 0.044).

Despite the substantial improvements in multiple parameters, the reduction in urine protein abnormalities was more gradual in both groups, with 78.6% of telitacicept patients and 73.6% of belimumab patients still showing abnormal values at 6 months (Figure 3).

### Safety

Both treatment regimens were generally well-tolerated. Due to the limited sample size and observation period, a comprehensive safety analysis could not be conducted. No unexpected safety signals were observed in either group during the study period.

## Discussion

This real-world observational study provides preliminary evidence supporting the potential value of BAFF/APRIL expression testing in guiding treatment selection for SLE patients. Our findings suggest that a biomarker-guided approach using telitacicept for BAFF/APRIL double-positive patients may yield superior clinical outcomes compared to conventional treatment with belimumab without prior expression testing.

The most striking finding of our study is that despite having more severe baseline disease activity and more aggressive lupus nephritis, patients in the BAFF/APRIL-guided telitacicept group achieved comparable or better clinical responses than those in the conventional belimumab group. The telitacicept group demonstrated higher complete and partial response rates (57.1 and 35.7%, respectively), with a notably lower non-response rate (7.1% vs. 23.6%). This is particularly remarkable considering that the telitacicept group had significantly higher initial SLEDAI scores (18.79 vs. 8.86) and more severe proliferative lupus nephritis with greater histopathological activity.

The mechanistic basis for these findings likely relates to the differential targeting of B cell pathways by telitacicept and belimumab. Telitacicept is a fusion protein that combines the extracellular domain of TACI with the Fc fragment of human IgG1, enabling it to target both BAFF (BLyS) and APRIL simultaneously. This prevents their interaction with all their B cell ligands, including BCMA, which plays a crucial role in the survival of long-lived bone marrow plasma cells and plasmablasts (19). In contrast, belimumab selectively targets only BAFF, leaving the APRIL signaling pathway intact.

The APRIL-BCMA axis is particularly important in later stages of B cell differentiation and plasma cell survival. APRIL has a high-affinity bond with BCMA, while BAFF has a weaker interaction, suggesting that the APRIL-BCMA axis could dominate or partially substitute for reliance on BAFF during plasma cell development (20). Previous studies have shown that inhibition of both APRIL and BCMA can suppress plasma cell formation and autoantibody secretion more effectively than targeting BAFF alone (21). This dual-targeting mechanism may explain the more profound immunological improvements observed in our BAFF/APRIL-guided telitacicept group.

Indeed, the immunological improvement was substantially more pronounced in the BAFF/APRIL-guided group, with more rapid and complete normalization of complement C3 levels and anti-dsDNA antibodies. By 6 months, only 7.1% of patients in the telitacicept group still had abnormal anti-dsDNA antibodies, compared to 32.5% in the belimumab group. This substantial reduction in autoantibodies supports the hypothesis that dual targeting of BAFF and APRIL provides more comprehensive suppression of pathogenic B cell responses in patients with confirmed expression of these cytokines.

Another important finding was the superior steroid-sparing effect observed in the BAFF/APRIL-guided group. By 6 months, patients in this group achieved a significantly greater reduction in corticosteroid dose compared to the conventional treatment group (Δ = −35.00 vs −25.00 mg, *p* = 0.014). This is clinically relevant as reducing corticosteroid exposure is a key goal in SLE management to minimize long-term complications.

Our results should be contextualized within the broader landscape of BAFF/APRIL-targeting therapies. In international trials of belimumab (BLISS-52 and BLISS-76),12,13 SRI-4 response rates at week 52 ranged from 43.2 to 58% for belimumab compared to 33.5–44% for placebo. Another dual BAFF/APRIL inhibitor, atacicept, has also been studied in SLE with SRI-4 response rates at week 24 ranging from 53.8 to 57.8% versus 44.0% in placebo groups (22). However, early atacicept trials faced safety challenges, with some studies terminated prematurely due to serious adverse events, possibly related to high-dose regimens, concomitant immunosuppression, or atacicept’s particularly high affinity for APRIL (23–25). In contrast, our experience with telitacicept revealed no unexpected safety signals, though our sample size and follow-up duration were limited.

Our approach aligns with the emerging paradigm of precision medicine in autoimmune diseases. While SLE has traditionally been treated with relatively standardized approaches based primarily on clinical phenotypes rather than underlying biological mechanisms, our findings suggest that incorporating biomarker testing into treatment decisions may enhance therapeutic outcomes. The personalized approach based on BAFF/APRIL expression testing represents a step toward more individualized SLE management, similar to advances already achieved in oncology and other fields.

This study represents one of the early attempts to evaluate BAFF/APRIL expression-guided biological agent selection in a real-world setting. Previous clinical trials have demonstrated the efficacy of both belimumab and telitacicept in SLE, but they have typically not incorporated biomarker-based patient selection. Our approach of using tissue expression of BAFF/APRIL to guide therapy selection provides a novel framework for personalized SLE management that warrants further investigation.

Several limitations of our study should be acknowledged. First, the retrospective, observational design introduces potential selection bias and confounding factors. Second, the lack of formal sample size calculation and the substantially smaller telitacicept group (*n* = 14 vs. *n* = 72) significantly limit statistical power for comparisons between groups. This sample size limitation was inherent to the real-world nature of our study, where patient numbers were determined by the availability of those meeting inclusion criteria and the feasibility of conducting BAFF/APRIL expression testing. The small sample size particularly affects the reliability of subgroup analyses and limits the generalizability of our findings. Third, the non-randomized nature of treatment allocation means that unmeasured confounding factors may have influenced outcomes. Fourth, BAFF/APRIL expression was only assessed in the telitacicept group, so we cannot determine how many patients in the belimumab group might have been BAFF/APRIL positive. Fifth, the follow-up period of 6 months is relatively short for a chronic disease like SLE, and longer-term outcomes remain to be determined.

It is important to recognize that our findings represent preliminary evidence rather than definitive proof of the superiority of a biomarker-guided approach. While the results are encouraging, causal relationships between BAFF/APRIL expression, treatment selection, and clinical outcomes cannot be firmly established from this exploratory study. The clinical benefit observed in the BAFF/APRIL-guided telitacicept group might be due to the intrinsic superiority of telitacicept over belimumab in certain patient populations, independent of biomarker status.

Future research directions should include prospective randomized controlled trials comparing biomarker-guided versus conventional treatment approaches in SLE. Such studies should incorporate BAFF/APRIL testing in all patients to determine the predictive value of these biomarkers for response to different targeted therapies. Additionally, longer follow-up periods are needed to assess the durability of responses and long-term safety profiles. Exploration of other potential biomarkers that could complement BAFF/APRIL testing in guiding treatment decisions would also be valuable.

The integration of BAFF/APRIL testing into routine clinical practice would require standardization of assessment methods, determination of clinically relevant cutoff values, and cost-effectiveness analyses. Moreover, the accessibility of renal biopsy specimens for biomarker testing may be limited in some clinical settings, suggesting a need to explore less invasive biomarker sources such as serum or urine.

In conclusion, our study provides preliminary evidence supporting the potential value of BAFF/APRIL expression testing in guiding personalized treatment selection for SLE patients. The biomarker-guided approach using telitacicept for BAFF/APRIL double-positive patients demonstrated promising efficacy despite treating a population with more severe disease. While these findings require validation in larger, prospective studies, they suggest a path toward more individualized SLE management that could enhance treatment outcomes and patient quality of life.

## Conclusion

BAFF/APRIL expression-guided telitacicept therapy demonstrated superior efficacy in SLE patients with more severe baseline disease compared to conventional belimumab therapy. This personalized approach resulted in higher response rates, more substantial immunological improvements, and better steroid-sparing effects. Our findings suggest that BAFF/APRIL expression testing may represent a valuable biomarker for guiding treatment selection in SLE, potentially leading to more individualized and effective therapeutic strategies. These preliminary results warrant further investigation in larger, prospective studies to validate the clinical utility of BAFF/APRIL-guided treatment approaches.

## Data Availability

The datasets presented in this article are not readily available because our data availability statement indicates that data are available from the corresponding author upon reasonable request rather than being publicly deposited because the dataset contains sensitive patient information that requires protection under privacy regulations. The clinical and laboratory data collected include detailed medical records from a relatively small patient cohort that could potentially be identifiable even after standard de-identification procedures. Additionally, our study was conducted under ethics approval that specified data sharing limitations to protect patient confidentiality. We are committed to scientific transparency and will promptly evaluate and respond to reasonable requests from qualified researchers for specific data needed to verify our findings, subject to appropriate data sharing agreements that maintain patient privacy protections. Requests to access the datasets should be directed to Xiaopeng Tang, txp12002@outlook.com.
